# Enhancing immune response, antioxidant capacity, and gut health in growing beagles through a chitooligosaccharide diet

**DOI:** 10.3389/fvets.2023.1283248

**Published:** 2024-01-11

**Authors:** Guoqiang Cheng, Tingting Hu, Yu Zeng, Liangchun Yan, Yanglu Liu, Yongjin Wang, JieYing Xia, Han Dong, Dong Chen, Tingting Cheng, Guangneng Peng, Li Zhang

**Affiliations:** ^1^Sichuan Academy of Chinese Medicine Sciences, Chengdu, China; ^2^The Key Laboratory of Animal Disease and Human Health of Sichuan Province, College of Veterinary Medicine, Sichuan Agricultural University, Chengdu, China; ^3^Sichuan Center for Animal Disease Control and Prevention, Chengdu, China

**Keywords:** nutraceutical, immune response, antioxidant, intestinal microbiota, chitooligosaccharides, canines

## Abstract

Chitooligosaccharides (COS) have attracted significant attention due to their unique biological activities, water solubility, and absorbable properties. The objective of the present study was to investigate the impact of COS-supplemented diets on the immune response, antioxidative capacity, hematology, serum biochemistry, and modulation of intestinal microbiota in growing beagles. Twelve weaning male beagles (6 weeks old; weighing 3.6 ± 0.6 kg) were fed either a control diet (food without COS, *n* = 6) or a COS-supplemented diet (*n* = 6) twice daily for 7 weeks. Blood samples collected at weeks 4 and 7 indicated that hematology and serum biochemistry remained unaffected by COS supplementation. Compared with the control group, the test group showed higher levels of serum antibodies against the canine distemper virus and parvovirus, higher levels of immunoglobulin A, G, and M, and increased activities of superoxide dismutase, glutathione peroxidase, and catalase. In addition, COS was observed to modulate the intestinal flora by enhancing the presence of probiotics, such as *Muribaculaceae*, *Prevotellaceae_Ga6A1_group*, *Lactobacillus*, *Collinsella*, *Blautia*, and *Lachnospiraceae_NK4A136_group*. In summary, a COS-supplemented diet could effectively improve dog health by regulating immune function and antioxidant responses and modulating intestinal microbiota. This study highlights the potentiality of using COS as a valuable nutraceutical for growing dogs.

## Introduction

The global population of companion animals is constantly increasing, with the number of domestic dogs currently estimated to be approximately 900 million worldwide (1). The growing number of domestic dogs has raised concerns regarding the safety and nutritional value of pet food (2). In the past, dogs were primarily used as watchdogs, shepherds, or hunters. In contrast, domestic dogs are nowadays largely kept as household pets instead of working animals. Therefore, dog owners often seek diets that can meet their dog’s basic needs and provide added benefits, such as enhanced immunity (3).

Chitooligosaccharides (COS) are low-molecular-weight derivatives of chitosan (4). Chitosan is a biocompatible cationic polysaccharide that was discovered in the late 1850s (5). Commonly encountered in nature, chitosan is produced by the deacetylation of chitin, which is naturally found in crustacean shells (6). While chitosan has received significant attention in various industries, including biomedicine (7), food (8), and cosmetics (9), its application is limited by its unfavorable properties, such as water insolubility and large molecular weight. Hence, COS are considered more suitable for various applications due to their superior water solubility, lower viscosity, and relatively small molecular size (5).

Chitooligosaccharides consist of acetylglucosamine (GlcNAc) or glucosamine (GlcN) units linked by β-1, 4-O-glycoside bonds (10), generated by the enzymatic or chemical hydrolysis of chitosan (11). Previous toxicological studies showed that COS were almost completely nontoxic following an acute intake administered at a maximum concentration of >10 g/kg in mice and a subchronic intake of 3.0 g/kg per day for 30 days in rats (12). Owing to their small molecular size, COS can be easily absorbed in the intestines and transported in blood, subsequently inducing systemic effects (5). COS exhibit a range of biological functions, including antimicrobial, anti-inflammatory, antioxidant, anti-tumor, immune-stimulating, and cholesterol-lowering activities (13).

Chitooligosaccharides supplementation in animal diets is harmless to the physical condition of the animals. Earlier studies on layer and broiler chicken models revealed that red blood cell (RBC) and white blood cell (WBC) counts were elevated as a result of COS supplementation. In addition, serum iron concentrations were also increased, facilitating improved iron utilization (14), egg weight, eggshell quality, and immunity in Hy-Line brown layers (15). Similar results were also obtained from studies on pigs (16–18) and tilapia (19). COS can actively participate in regulating the metabolism of nutrients, and play a good role in maintaining the health of pets. The application of COS in pet food can increase the number and flora of beneficial microorganisms, and inhibit harmful intestinal microorganisms, so as to promote the ecological balance of normal flora in the intestine and effectively eliminate the adverse effects of harmful microorganisms on pets. Therefore, in the field of animal husbandry and veterinary medicine, COS, as a natural active substance for immune enhancement and inflammation regulation, has high research value and broad application prospects in enhancing animal immune function and anti-inflammatory.

Immunity plays an important role in animal health. Immunostimulating medicines and dietary additives have been extensively investigated to enhance immune function. Studies have demonstrated that COS could stimulate the immune system through their interactions with membrane receptors on the surface of toll-like receptor 4-dependent macrophages (20), thereby promoting transcription and secretion of cytokines (21). Other immune effects include the promotion of IKKβ, TRAF6, and JNK1 gene expression (key molecules of the NF-κB and AP-1 pathways) and induction of the phosphorylation of IκBα in RAW264.7 macrophages (22). Furthermore, COS administration significantly increases the mRNA expression of COX-2, IL-10, and McP-1 (*p* < 0.05). These results suggest that COS can induce immunomodulatory responses (22) and other immunostimulatory effects (10).

Oxidative stress is a threat to animal health, especially for growing and/or active animals (23). Oxidative stress results from the accumulation of reactive oxygen and reactive nitrogen species (24). Previous studies demonstrated that exercise-induced oxidative stress results in increased muscle fatigue, muscle fiber damage (25), and damage to the immune system (26). To counteract these effects, antioxidant enzymes, including superoxide dismutase (SOD), glutathione peroxidase (GSH-Px), and catalase (CAT), become available in both blood and skeletal muscles (9, 27, 28). Chitosan and its derivatives have been shown to possess scavenging potential and inhibit oxidative damage by blocking free radical chain reactions (10). Importantly, it has previously been shown that COS and their derivatives exhibit stronger antioxidant activities than chitosan (29).

Intestinal health is not only reflected in the integrity of structure and function of the intestinal microecological environment, but also in its stability. Previous research studies indicated that incorporating prebiotics such as oligofructose, hemicellulose, and pectin into the diets of cats and dogs could yield noteworthy benefits (30–32). This dietary modification elevates the presence of beneficial bacteria such as *Lactobacilli* and *Bifidobacteria*, while concurrently diminishing the population of detrimental microorganisms such as enterotoxin-producing *Escherichia coli* and *Clostridium perfringens* (33). The outcomes of a previous study on juvenile Tibetan mastiffs further corroborate this notion. The authors demonstrated that oligosaccharides play a pivotal role in fostering a harmonious intestinal flora balance, thereby mitigating diarrhea and improving fecal quality among young Tibetan mastiffs (34).

As discussed above, COS as a novel type of harmless additive, exhibit good antioxidant properties, immune-regulation capabilities, and effects in improving intestinal microecological balance. Moreover, they do not exert any toxic side effects on the animal body, rendering these chitosan derivatives safe and reliable. However, their effect on dogs has not been investigated yet. Since COS are a relatively new and uncommonly used canine dietary supplement, their effects on the health of dogs need to be evaluated. Therefore, this randomized controlled study sought to determine the effect of dietary supplementation with COS on the immunity, antioxidative capacity, and intestinal microbiota in growing beagles.

## Materials and methods

### Animals

A group of 12 male weaning beagles (6 weeks old; weighing 3.6 ± 0.6 kg) were purchased from the Beagles Breeding Center (Sichuan Institution of Musk Deer Breeding, Sichuan, China) and randomly divided into two experimental groups (*n* = 6 each). One group (S) received a COS-supplemented diet, whereas the second group (C) served as the control. All dogs were examined by a veterinarian from the veterinary hospital of Sichuan Agricultural University, where the study was conducted, and found to be healthy. All dogs were reared for 2 weeks to help them adapt to the experimental conditions. All procedures used in the present study were approved by the Animal Care Advisory Committee of the Sichuan Academy of Chinese Medicine Sciences (SCACMS-20180406).

### Feeding trial

**Figure 1:**
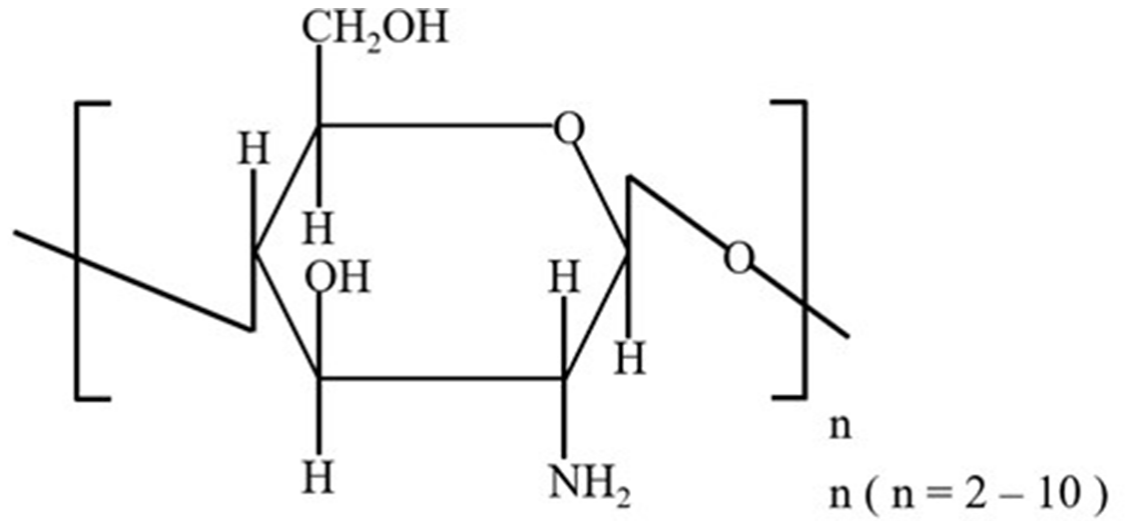
Structural formula of the COS used in this study.

During the 2-week acclimation period, all dogs were vaccinated (VANGUARD® PLUS 5, SmithKline Beecham, West Chester, PA, United States), with vaccine boosters administered at weeks 2 and 5 of the feeding trial. After 2 weeks of acclimation, the 7-week feeding trial began. Dogs were housed in separate kennels (1.5 m × 1.0 m) in a climate-controlled room (24°C ± 2°C) with a 12-h light/dark cycle. Each kennel was equipped with a feeder and a water bucket, and fresh water was provided *ad libitum*. To meet their metabolic energy requirements (35), dogs were individually fed twice a day (9.00 AM and 6.00 PM) with a diet comprising basic, nutritionally complete, and extruded dry dog food (Steak & Fruits & Vegetables Flavor Complete Food for Puppy, Eple, NORY®, Brige PetCare Co., Shanghai, China), consisting of 22.0% crude protein, 11.6% crude fat, 5.0% crude fiber, 10.0% crude ash, 0.77% lysine, 1.2% calcium, 1.0% phosphorus, 10.0% moisture, and 0.45% water-soluble chloride. COS in powder form were added to the food using a mixer (Group S: 0.5%, Group C: 0%). The additive amount of 0.5% COS was selected in the formal experiment. For the reason, firstly, we have consulted many references and the same dose have been used in these studies (36–38). At the same time, our pre-experiment also indicated the additive amount was a safe and effective dose. Dogs were weighed weekly throughout the trial. COS used in this study, with an average molecular weight of <1,000, a polymerization degree of 2–7, and > 90% purity, were purchased from the Dalian Institute of Physical Chemistry, Chinese Academy of Sciences, Dalian, China, and were prepared using enzyme reaction-membrane separation coupling technology (39). The structural formula of oligomeric β-(1–4)-2-amino-2-deoxy-D-glucose is shown in Figure 1.

### Blood sampling and analysis

Blood samples were collected on weeks 4 and 7 after a 12-h fast. Three types of vacutainers were used to collect blood, namely two vacutainers containing either EDTA-K2 or heparin lithium, and a third vacutainer which did not contain any additives. Tubes containing heparin lithium and those with no additives were then centrifuged (2,500 rpm, 4°C, 10 min) to obtain serum, followed by storage at −80°C.

The following hematological parameters were analyzed using the URIT-5160 5-Part-Diff Hematology Analyzer (URIT Medical Electronic CO., LTD. Guilin, China): total WBC count, total lymphocyte, monocyte, neutrophil, eosinophil, and basophil counts and ratios, total RBC count, RBC distribution width, mean corpuscular volume, mean hemoglobin content and concentration, hematocrit, total platelet count, mean platelet volume, platelet distribution width, large platelet ratio, and numbers and percentages of abnormal lymphocytes, giant immature cells, and nucleated RBCs.

The parameters used to evaluate serum biochemical profiles included albumin, alkaline phosphatase, alanine aminotransferase, amylase, blood urea nitrogen, calcium, cholesterol, creatinine, gamma-glutamyl transferase, globulin, glucose, lipase, phosphorus, total bilirubin, total protein, total protein/globulin, and blood urea nitrogen/creatinine ratios. All parameters were analyzed using an IDEXX Catalyst One Analyzer (IDEXX Laboratories, Inc., Westbrook, ME, United States). The levels of immunoglobulin A (IgA), immunoglobulin G (IgG), immunoglobulin M (IgM), canine distemper virus-antibody (CDV-Ab), and canine parvovirus-antibody (CPV-Ab) in the serum were assessed using commercial ELISA kits (Shanghai Yu Bo Biotech Co., Ltd., Shanghai, China). The levels of SOD, GSH-Px, and CAT were also evaluated using ELISA kits (Shanghai FANKEL Industrial Co., Ltd., Shanghai, China).

### 16S rRNA sequencing

Fecal samples were snap frozen and stored at −80°C after collection. To profile potential alterations in the gut microbiome after COS exposure, 16S rRNA sequencing was performed. Briefly, total fecal DNA was isolated using the MagPure Soil DNA LQ Kit (Magen, Guangdong, China). DNA concentration and integrity were measured by a NanoDrop 2000 spectrophotometer (Thermo Fisher Scientific, Waltham, MA, United States) and agarose gel electrophoresis, respectively. PCR amplification of the V3-V4 hypervariable regions of the bacterial 16S rRNA gene was carried out using universal primer pairs (343F: 5′-TACGGRAGGCAGCAG-3; 798R: 5′-AGGGTATCTAATCCT-3′). The reverse primer contained a sample barcode, and both primers were connected with an Illumina sequencing adapter. The PCR products were purified with Agencourt AMPure XP beads (Beckman Coulter Co., United States) and quantified using Qubit dsDNA assay kit. The concentrations were then adjusted for sequencing, which was performed on Illumina NovaSeq6000 with two paired-end read cycles of 250 bases each (Illumina Inc., San Diego, CA, United States; OE Biotech Company; Shanghai, China).

The raw reads were filtered and merged by Trimmomatic and FLASH software to obtain high-quality sequencing reads. Then, reads with chimera were detected and removed using VSEARCH. Clean reads were subjected to primer sequences removal and clustering to generate operational taxonomic units (OTUs) using VSEARCH software with 97% similarity cutoff. The representative read of each OTU was selected using the QIIME package. All representative reads were annotated and blasted against Silva database (*v*. 132) using an RDP classifier (confidence threshold was 70%).The 16S rRNA gene amplicon sequencing and analysis were conducted by OE Biotech Co., Ltd. (Shanghai, China).

### Statistical analysis

Statistical analysis was performed using SPSS *v*.22.0 (IBM Corp., Armonk, NY, United States). Differences in data between the two groups were analyzed using a two-tailed *t*-test, and differences between groups were considered statistically significant when *p* < 0.05.

## Results

### COS improved the body weight gain of dogs

**Figure 2:**
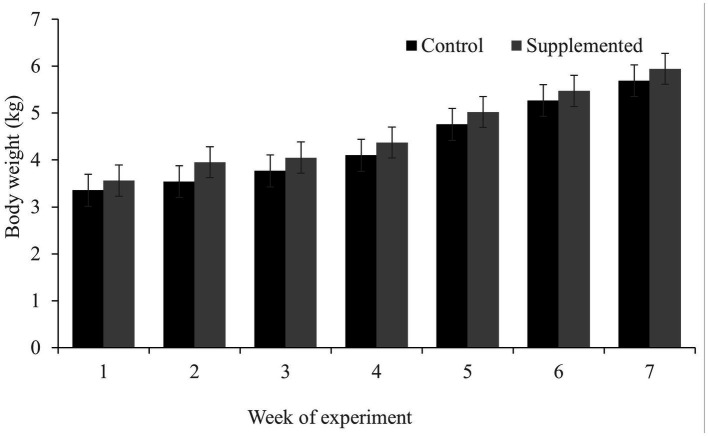
Body weight of dogs in the COS-supplemented (*n* = 6) and control groups (*n* = 6). Differences were non-significant. The results are presented as the mean ± SD.

While both groups gained weight during the feeding trial (which is expected in dogs during the growth phase; Figure 2), no significant differences were observed in the body weight between the two groups at the time points examined (*p* > 0.05). Throughout the experiment, all dogs were found to be in good physical condition and well-fed. No side effects of diarrhea were observed at any point during the study.

### COS had no impact on hematology and blood biochemical indicators of dogs

Hematology results revealed no defects in either group throughout the study (*p* > 0.05, Tables 1, 2). Moreover, there were no significant differences in serum biochemistry between groups (*p* > 0.05, Tables 3, 4).

**Table 1:** Blood hematology parameters of dogs fed with control and supplemented diets at Week 1 (values are presented as mean ± SD).

Item	Unit	Control group (*n* = 6)	Supplemented group (*n* = 6)	*p* value
WBC	10^9^/L	10.45 ± 1.23	10.35 ± 1.73	0.91
LYM%	%	23.75 ± 9.25	26.34 ± 4.53	0.55
MON%	%	11.38 ± 1.76	9.88 ± 2.61	0.27
NEU%	%	63.70 ± 9.80	61.15 ± 4.96	0.58
EOS%	%	1.21 ± 0.25	1.65 ± 0.84	0.42
BASO%	%	0.48 ± 0.25	0.40 ± 0.04	0.58
LYM	10^9^/L	2.40 ± 0.68	2.79 ± 0.93	0.43
MON	10^9^/L	1.18 ± 0.14	0.77 ± 0.10	0.25
NEU	10^9^/L	7.03 ± 2.62	7.58 ± 0.55	0.52
EOS	10^9^/L	0.10 ± 0.05	0.17 ± 0.08	0.17
BASO	10^9^/L	0.05 ± 0.03	0.04 ± 0.01	0.46
RBC	10^12^/L	6.77 ± 1.12	5.90 ± 0.38	0.10
HGB	g/L	145.66 ± 25.46	134.00 ± 8.17	0.31
HCT	%	40.48 ± 7.70	37.01 ± 2.61	0.30
MCV	fL	60.03 ± 5.50	62.80 ± 1.37	0.26
MCH	Pg	21.57 ± 2.31	22.70 ± 0.71	0.27
MCHC	g/L	371.33 ± 14.37	367.33 ± 6.65	0.68
RDW	%	15.65 ± 2.50	13.60 ± 0.61	0.08
CV RDW-CV	%	15.65 ± 2.50	13.60 ± 0.61	0.08
SD RDW-SD	fL	34.53 ± 1.15	33.48 ± 0.78	0.10
PLT	10^9^/L	338.67 ± 15.57	416.50 ± 80.37	0.15
MPV	fL	9.70 ± 0.55	10.58 ± 0.79	0.05
PDW	fL	14.18 ± 1.24	15.85 ± 2.31	0.15
PCT	%	0.30 ± 1.17	0.43 ± 0.09	0.13
P-LCR	%	35.18 ± 9.79	37.00 ± 3.81	0.07
P-LCC	10^9^/L	135.50 ± 49.76	163.83 ± 43.16	0.37
ALY%	%	0.52 ± 0.34	0.45 ± 0.18	0.68
LIC%	%	0.96 ± 0.51	0.78 ± 0.38	0.55
NRBC%	%	8.42 ± 3.70	8.70 ± 2.96	0.49
ALY	10^9^/L	0.05 ± 0.03	0.08 ± 0.03	0.84
LIC	10^9^/L	0.09 ± 0.06	0.04 ± 0.02	0.11
NRBC	10^9^/L	0.88 ± 0.37	0.76 ± 0.23	0.52

WBC, White blood cell count; LYM%, Lymphocyte ratio; MON%, Monocyte ratio; NEU%, Neutrophil ratio; EOS%, Eosinophil ratio; BASO%, Basophil ratio; LYM, Total lymphocyte count; MON, Total number of monocytes; NEU, Neutrophil count; EOS, Eosinophil count; BASO, Basophil count; RBC, Total red blood cell count; HGb, Hemoglobin; HCT, Hematocrit; MCV, Mean corpuscular volume; MCH, Mean hemoglobin content; MCHC, Mean hemoglobin concentration; RDW, Red blood cell distribution width; CV RDW-CV, Red blood cell distribution width; SD RDW, Red blood cell distribution width; PLT, Total platelet count; MPV, Mean platelet volume; PDW, Platelet distribution width; PCT, Platelet count; P-LCR, Large platelet ratio; P-LCC, Total platelet count; ALY%, Percentage of abnormal lymphocytes; LIC%, Percentage of giant immature cells; NRBC%, Percentage of nucleated red blood cells; ALY, Number of abnormal lymphocytes; LIC, Number of large immature cells; and NRBC, Number of nucleated red blood cells.

**Table 2:** Blood hematology parameters of dogs fed with control and supplemented diets at Week 7 (values are presented as mean ± SD).

Item	Unit	Control group (*n* = 6)	Supplemented group (n = 6)	*p* value
WBC	10^9^/L	10.00 ± 2.31	8.81 ± 1.26	0.30
LYM%	%	20.48 ± 2.86	2457.00 ± 4.57	0.07
MON%	%	10.23 ± 0.81	8.96 ± 1.95	0.17
NEU%	%	67.13 ± 2.97	63.34 ± 6.37	0.22
EOS%	%	1.96 ± 0.76	1.46 ± 0.43	0.19
BASO%	%	0.20 ± 0.11	0.17 ± 0.06	0.59
LYM	10^9^/L	2.02 ± 0.40	2.28 ± 0.40	0.30
MON	10^9^/L	1.02 ± 0.25	0.94 ± 0.10	0.67
NEU	10^9^/L	6.74 ± 1.76	5.62 ± 0.22	0.22
EOS	10^9^/L	0.19 ± 0.07	0.12 ± 0.03	0.11
BASO	10^9^/L	0.02 ± 0.01	0.01 ± 0.00	0.40
RBC	10^12^/L	6.01 ± 0.56	5.76 ± 0.44	0.39
HGB	g/L	133.67 ± 4.51	133.33 ± 9.27	0.95
HCT	%	36.40 ± 2.50	36.01 ± 2.78	0.81
MCV	fL	60.90 ± 4.68	62.71 ± 1.47	0.39
MCH	Pg	22.27 ± 1.74	23.13 ± 0.65	0.28
MCHC	g/L	366.67 ± 4.50	371.00 ± 4.90	0.25
RDW	%	17.56 ± 5.58	13.77 ± 0.35	0.13
CV RDW-CV	%	17.56 ± 5.58	13.77 ± 0.35	0.13
SD RDW-SD	fL	36.51 ± 1.94	34.70 ± 0.47	0.16
PLT	10^9^/L	349.00 ± 32.40	334.83 ± 43.25	0.54
MPV	fL	9.33 ± 0.71	10.31 ± 0.13	0.14
PDW	fL	13.43 ± 0.99	15.55 ± 3.53	0.19
PCT	%	0.32 ± 0.03	0.33 ± 0.02	0.36
P-LCR	%	30.04 ± 5.23	36.25 ± 11.06	0.24
P-LCC	10^9^/L	103.50 ± 15.18	117.50 ± 25.41	0.27
ALY%	%	0.28 ± 0.13	0.32 ± 0.13	0.67
LIC%	%	0.53 ± 0.45	0.33 ± 0.23	0.35
NRBC%	%	8.56 ± 1.77	8.70 ± 2.97	0.93
ALY	10^9^/L	0.02 ± 0.01	0.03 ± 0.01	0.50
LIC	10^9^/L	0.05 ± 0.05	0.03 ± 0.02	0.35
NRBC	10^9^/L	0.85 ± 0.25	0.75 ± 0.33	0.65

ALB, Albumin; ALKP, Alkaline phosphatase; ALT, Alanine aminotransferase; AMYL, Amylase; BUN, Blood urea nitrogen; CA, Calcium ion; CHOL, Cholesterol; CREA, Creatinine; GGT, Gamma-glutamyl transferase; GLOB, Globulin; GLU, Blood glucose; LIPA, Lipase; PHOS, Phosphorus ion; TBIL, Total bilirubin; TP, Total protein; ALB/GLOB, Total protein/globulin ratio; and BUN/CREA, Blood urea nitrogen/creatinine ratio.

**Table 3:** Serum biochemical traits of dogs fed with control and supplemented diets at Week 4 (values are presented as mean ± SD).

Item	Unit	Control group (*n* = 6)	Supplemented group (*n* = 6)	*p* value
ALB	g/dL	2.64 ± 0.08	2.52 ± 0.11	0.07
ALKP	U/L	235.84 ± 23.67	261.34 ± 46.05	0.26
ALT	U/L	29.84 ± 18.22	33.00 ± 6.41	0.70
AMYL	U/L	478.34 ± 149.48	451.84 ± 150.24	0.77
BUN	mg/dL	7.17 ± 3.76	6.00 ± 0.90	0.48
CA	mg/dL	10.93 ± 0.14	10.68 ± 0.38	0.16
CH0L	mg/dL	162.00 ± 13.20	174.33 ± 26.60	0.33
CREA	mg/dL	0.32 ± 0.08	0.32 ± 0.08	1.00
GGT	U/L	1.83 ± 4.49	0.17 ± 0.41	0.39
GLOB	g/dL	2.75 ± 0.06	2.72 ± 0.17	0.66
GLU	mg/dL	99.33 ± 5.57	100.33 ± 5.61	0.76
LIPA	U/L	581.83 ± 82.14	550.50 ± 95.94	0.56
PHOS	mg/dL	7.17 ± 0.43	7.63 ± 0.43	0.09
TBIL	mg/dL	0.12 ± 0.04	0.17 ± 0.04	0.04
TP	g/dL	5.38 ± 0.08	5.23 ± 0.27	0.21
ALB/GLOB		0.98 ± 0.04	0.93 ± 0.05	0.09
BUN/CREA		0.98 ± 0.17	0.98 ± 0.08	0.33

**Table 4:** Serum biochemical traits of dogs fed with control and supplemented diets at Week 7 (Values are presented as mean ± SD).

Item	Unit	Control group (*n* = 6)	Supplemented group (*n* = 6)	*p* value
ALB	g/dL	2.61 ± 0.26	2.57 ± 0.08	0.67
ALKP	U/L	169.67 ± 45.89	200.83 ± 64.30	0.36
ALT	U/L	35.00 ± 7.64	43.50 ± 17.67	0.31
AMYL	U/L	639.00 ± 183.62	514.83 ± 103.52	0.18
BUN	mg/dL	8.83 ± 2.72	7.33 ± 1.51	0.26
CA	mg/dL	7.05 ± 4.69	7.02 ± 4.66	0.10
CH0L	mg/dL	160.33 ± 0.08	175.50 ± 14.69	0.15
CREA	mg/dL	0.37 ± 0.08	0.40 ± 0.09	0.45
GGT	U/L	0.33 ± 0.81	1.33 ± 2.06	0.30
GLOB	g/dL	2.68 ± 0.15	2.58 ± 0.10	0.20
GLU	mg/dL	91.83 ± 4.17	88.00 ± 5.18	0.19
LIPA	U/L	659.67 ± 82.14	497.33 ± 73.28	0.14
PHOS	mg/dL	6.98 ± 0.30	6.77 ± 0.44	0.35
TBIL	mg/dL	0.10 ± 0.00	0.10 ± 0.00	1.00
TP	g/dL	5.30 ± 0.17	5.15 ± 0.08	0.08
ALB/GLOB		0.98 ± 0.17	0.98 ± 0.08	1.00
BUN/CREA		25.00 ± 10.26	19.00 ± 5.55	0.24

### COS significantly improved the immune response of dogs

**Figure 3:**
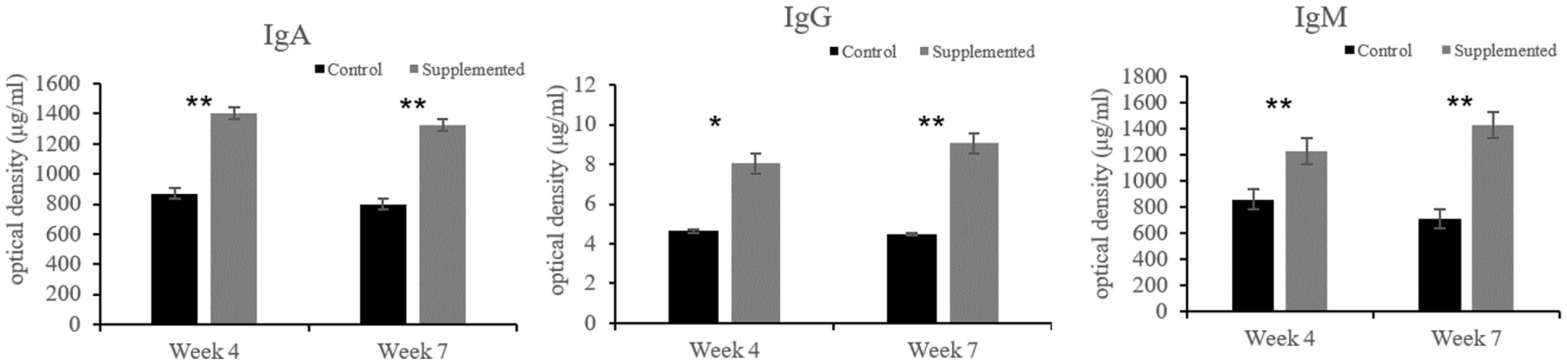
Concentrations of immunoglobulin (Ig)A, G, and M, in COS-supplemented and control groups. ^*^p < 0.05; ^**^p < 0.01. Results are presented as the mean ± SD (*n* = 6).

**Figure 4:**
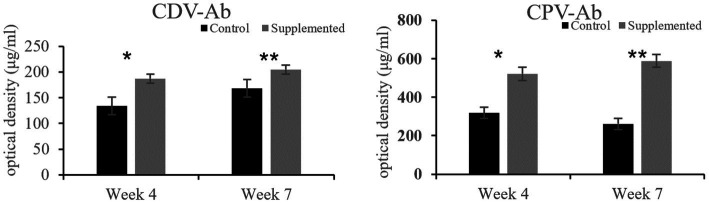
Concentrations of antibodies against canine distemper virus (CDV-Ab) and parvovirus (CPV-Ab) in COS-supplemented and control groups. ^*^p < 0.05; ^**^p < 0.01. Results are presented as the mean ± SD (*n* = 6).

Immunoglobulin A and IgG concentrations in the COS-supplemented group were significantly higher than those in the control group at weeks 4 (*p* = 0.004 and *p* = 0.02, respectively) and 7 (*p* = 0.0001 and *p* = 0.0003, respectively). Furthermore, IgM concentrations in the COS-supplemented group were significantly higher than those in the control group at weeks 4 (*p* = 0.0009) and 7 (*p* = 0.000004, Figure 3). Similarly, the concentrations of both CDV-Ab and CPV-Ab were also higher in the COS-supplemented group at weeks 4 and 7 (*p* < 0.05), as shown in Figure 4.

### COS upregulated the antioxidative capability of dogs

**Figure 5:**
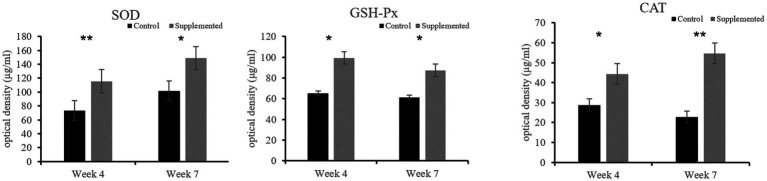
Activities of the antioxidant enzymes superoxide dismutase (SOD), glutathione peroxidase (GSH-Px), and catalase (CAT) in COS-supplemented and control groups. ^*^p < 0.05; ^**^p < 0.01. Results are presented as the mean ± SD (*n* = 6).

Both SOD and GSH-Px activities in the COS-supplemented group were higher than those in the control group at weeks 4 (*p* = 0.009 and *p* = 0.037, respectively) and 7 (*p* = 0.015 and *p* = 0.023, respectively). Furthermore, CAT activity in the COS-supplemented group was also significantly higher than that in the control group at weeks 4 (*p* < 0.05) and 7 (*p* < 0.001), as shown in Figure 5.

### COS improved the intestinal microbiota

**Figure 6:**
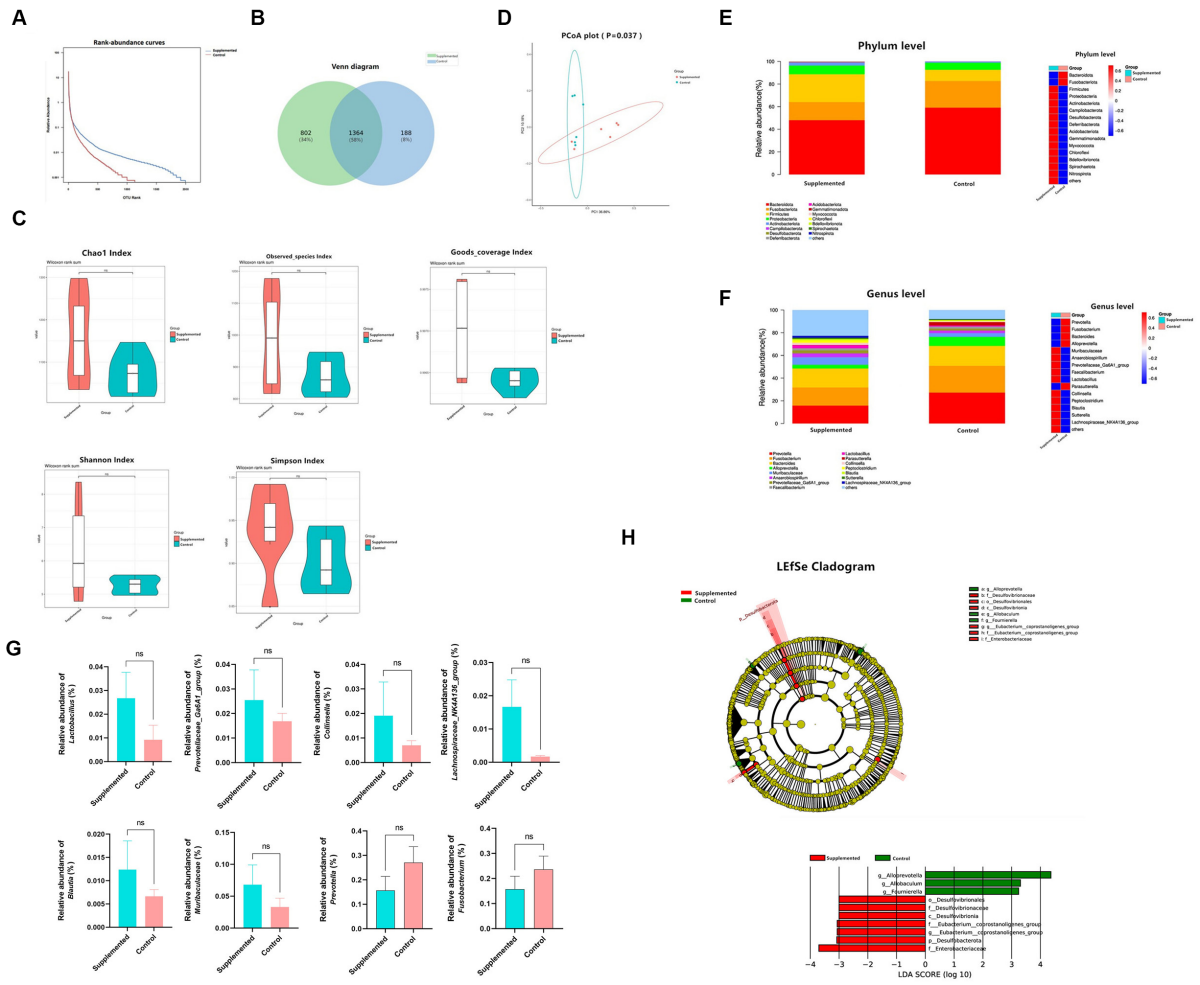
COS regulates gut microbiota. **(A)** Rank-abundance curves of all samples in the Supplemented and Control groups. The ordinate and the abscissa represent the relative percent content of the OTU number and the ranking level of the OTU number, respectively. **(B)** venn Polt. **(C)** Difference in alpha diversity between the Supplemented and the Control groups, demonstrated by Chao1, Observed_species, Goods_coverage, Shannon, and Simpson indexes. **(D)** Principal coordinate analysis (PCoA) for the composition of fecal samples. The red and green spots correspond to the Supplemented samples and the Control samples, respectively. **(E)** Differences in relative microbial abundance between the two groups at the Phylum level; bar plot on the left, heat map on the right. **(F)** Differences in relative microbial abundance between the two groups at the Genus level; bar plot on the left, heat map on the right. **(G)** Relative abundance of *Lactobacillus*, *Prevotellaceae_Ga6A1_group*, *Collinsella*, *Lachnospiraceae_NK4A136_group*, *Blautia*, *Muribaculaceae* and *Prevotella*, and *Fusobacterium*. Results are presented as mean values ± SEM (*n* = 6). **(H)** LEfSe cladogram indicating that multiple taxa from Phylum to Genus were differentially enriched in the corresponding groups. The size of the dots represents the abundance of microbiota. Wilcoxon rank-sum test (95% confidence interval) was performed to screen the differential microbiota. Species exhibiting LDA scores that exceed the predetermined significance thresholds were recognized as statistically distinct biomarkers. The colors of the bars on the chart correspond to their respective groups, while their length signifies the LDA scores, reflecting the degree of influence by significantly disparate species among various groups.

The 16S rRNA sequencing results showed a significant difference in the OTU abundance or evenness between the two groups (Figures 6A,B). With regard to alpha diversity, the COS-supplemented group exhibited increased abundance and evenness in the Observed_species, Chao1, Goods_coverage, Shannon, and Simpson indexes compared to the control group. In contrast, no statistically significant difference (*p* > 0.05) was observed in all groups (Figure 6C). With regard to beta diversity, according to the principal coordinate analysis (PCoA), the composition of fecal samples in the COS-supplemented group was significantly different from that in the control group (*p* < 0.05), and the samples of the COS-supplemented group exhibited a certain degree of intragroup similarity (Figure 6D).

In terms of species composition, at the Phylum level, the COS-supplemented group exhibited an increased relative abundance of Firmicutes and Actinobacteriota, and a reduced abundance of Bacteroidota and Fusobacteriota, whereas that of Proteobacteria remained unchanged (Figure 6E). At the Genus level, the relative abundance of *Lactobacillus* probiotics, *Prevotellaceae_Ga6A1_group*, *Collinsella*, *Lachnospiraceae_NK4A136_group*, *Blautia*, and *Muribaculaceae* were all significantly increased by COS, while harmful bacteria, such as *Prevotella* and *Fusobacterium*, were significantly reduced (Figures 6F,G).

Based on the linear discriminant analysis (LDA), 10 taxa were identified of having high effect sizes from Phylum to Genus level with the following parameters: value of *p* < 0.05, LDA SCORE (log 10) > 2. The species affiliation and corresponding taxonomic groups are displayed in the LEfSe cladogram shown in Figure 6H. These results indicate that COS has the potential to modulate gut microbiota and enhance the abundance of beneficial probiotics.

## Discussion

While previous animal-based studies confirmed the effectiveness of COS-supplemented diets on improving the health of various animals (14), the present study is the first to our knowledge to evaluate the effect of a COS-supplemented diet on the health of dogs. The biological and medicinal properties of COS as a potential nutraceutical, including their immunostimulatory and antioxidant functions, and their ability to regulate intestinal flora, are well-documented in literature (13). Similar to the findings of previous studies performed in non-canine species, our study demonstrated that a COS-supplemented diet could promote immunostimulatory and antioxidant activities, and also regulate the intestinal flora in dogs.

The effects of supplements on the general well-being of animals can be determined via physical examination and quantification of various hematological and serum biochemical parameters. Our results showed that there was no statistical difference in the hematological and serum parameters in both the control and COS-supplemented dog groups, which is consistent with previous results published on rats and mice (12, 40). Consequently, our findings suggest that a diet containing 0.5% COS has no negative effects on canine health.

The immune system plays an important role in eliminating foreign pathogens. Immunostimulatory medicines and nutraceuticals have attracted significant attention with respect to their potential for improving immune functions (10). Prior studies noted the potential of COS as immunostimulatory agents in mice (37). Our study sought to determine whether COS could function as immunostimulants when administered as canine dietary supplements. We found that a COS-supplemented diet improved the immune function of beagle dogs. As expected, following vaccination, IgA, IgG, IgM, CDV-Ab, and CPV-Ab levels in the COS-supplemented group were significantly higher than those in the control group. These results corroborate the findings of a previous study in which mice administered with COS via intraperitoneal injection exhibited increased secretion of interleukin-1, interleukin-2, and interferon-γ (41). A possible explanation for these results is that the positive charge that COS possess facilitates their combination or reaction with antigens, which subsequently increases their immunogenicity (42) and enhances their uptake by macrophages (43). Regarding the mechanism underlying the COS-enhanced ability of antigens to bind to components of the immune system, one may speculate that it is related to the COS-mediated activation of macrophages and dendritic cells through mannose receptors or toll-like receptor 4 (44). Therefore, this mechanism could be related to the induction of dendritic cell maturation and increased antigen capture and subsequent presentation. Similar to dendritic cells, macrophages are a critical part of the immune system, acting as a bridge to immune responses and promoting the secretion of antigens and immunoglobulins by B lymphocytes (45). Increased levels of specific antibodies and secretion of IgG and IgM were also observed in a mouse experiment in which COS were administered in combination with the porcine circovirus vaccine (46). Furthermore, dietary COS were shown to enhance the immune performance of broilers in response to the coccidia vaccine (47). Additionally, COS has been found to enhance immune protection have a formalin-inactivated *Vibrio anguillarum* vaccine in zebrafish and turbot (48).

While this study is the first to investigate whether supplementing canine diets with COS could facilitate the secretion of immune proteins, our results should be interpreted with caution due to the limited sample size used herein. Consequently, further research is required to elucidate the mechanisms whereby COS improve antibody responses, as observed in this study.

An additional objective of the present study was to evaluate the potential capacity of long-term dietary supplementation with antioxidants to regulate oxidative stress (49). Changes in the environment, temperature, exercise, and competition can all cause stress in dogs, which may contribute to the development of metabolic and degenerative diseases (50). The balance between oxidative stress generated by noxious free radicals and protective antioxidant molecules produced by continuously occurring metabolic oxidation reactions is critical to their overall health. The results of our study indicated that administration of COS-supplemented diets for a period of 7 weeks increased the activities of antioxidant enzymes SOD, GSH-Px, and CAT in canines. Therefore, the present study clearly demonstrates the ability of a COS-supplemented diet to reduce oxidative stress. Previous research studies have established that the free radical scavenging potential of COS is induced by interrupting free radical-producing chain reactions, thus inhibiting oxidative damage (29). Similarly, a study on mice fed with COS-supplemented diets showed significantly increased activities of SOD, GSH-Px, and CAT in the stomach, liver, and serum (38). However, the molecular mechanisms by which COS scavenge free radicals still remain unclear (51). This study thus lays the foundation for future investigations on using COS as an antioxidant dog food additive.

Chitooligosaccharides are usually defined as prebiotics that can selectively enhance the growth of beneficial bacteria. Li et al. validated through an *in vitro* MTT assay that COS exhibits notable proliferative effects on gut probiotics such as *Lactobacillus bulgaricus* and *Streptococcus thermophilus* (52). Ren et al. administered 600 mg/kg of COS to mice via gastric gavage for a period of 7 days. The analysis of alterations in the gut microbiota revealed that the group receiving chitosan oligosaccharides exhibited a significant increase in the quantity of *Bifidobacteria* compared to the control group (*p* < 0.05), while there was a decreasing trend in the populations of *Escherichia coli* and *Enterococcus* (53). The results of the present experimental study showed that dietary COS included in diets for 0.5% COS increased the abundance of the probiotics *Muribaculaceae*, *Prevotellaceae_Ga6A1_group*, *Lactobacillus*, *Collinsella*, *Blautia*, and *Lachnospiraceae_NK4A136_group*, and decreased the population of *Prevotella* and *Fusobacterium*. The study conducted by Yang et al. revealed that dietary supplementation of chitosan oligosaccharides (COS) at doses of 400 or 600 mg/kg enhanced growth performance, bolstered gut barrier function, amplified the populations of *Bifidobacteria* and *Lactobacilli*, and reduced the presence of *Staphylococcus aureus* in the cecum of weanling pigs (54).

In the present study, we concluded that growing beagles could ingest a 0.5% COS-supplemented diet without any negative effects on their hematology levels or serum biochemistry. Importantly, COS was found to enhance the activity of antioxidant enzymes and increase circulating antibody levels following vaccination against CDV and CPV in beagle dogs. Moreover, COS could regulate gut microbiota, indicating their potential to promote intestinal health to a certain extent. These findings suggest that COS could serve as a high-quality, immune-stimulating, antioxidative, and intestinal health-promoting nutraceutical for growing dogs. Notwithstanding the relatively limited sample size, this study presents valuable insights into the supplementation of diets with COS.

## Data availability statement

The original contributions presented in the study are publicly available. This data can be found in the repository: China National Centre for Bioinformation under accession number: CRA013776 and is available at this link: https://ngdc.cncb.ac.cn/search/?dbId=gsa&q=CRA013776&page=1.

## Ethics statement

The animal study was approved by the Animal Care Advisory Committee of the Sichuan Academy of Chinese Medicine Sciences. The study was conducted in accordance with the local legislation and institutional requirements.

## Author contributions

GC: Writing – original draft. TH: Writing – original draft. YZ: Data curation, Writing – original draft. LY: Methodology, Writing – original draft. YL: Methodology, Writing – original draft. YW: Data curation, Writing – original draft. JX: Data curation, Writing – original draft. HD: Methodology, Software, Writing – original draft. DC: Data curation, Software, Writing – original draft. TC: Software, Writing – original draft. GP: Writing – review & editing, Project administration. LZ: Writing – review & editing, Project administration.
